# Seasonal outdoor thermal comfort and neutral PET thresholds in a severe cold Dwa climate city of Shenyang China

**DOI:** 10.1038/s41598-026-46003-0

**Published:** 2026-04-01

**Authors:** Lei Fan, Zixian Li, Yan zhou

**Affiliations:** 1 Forestry College, Shenyang Agricultural University, Shenyang, 110866 China; 2 Key Laboratory of Northern Landscape Plants and Regional Landscape, Shenyang, 110866 China

**Keywords:** Physiologically equivalent temperature (PET), Thermal comfort, Cold climate, Sky view factor (SVF), Thermal adaptation behavior, Cold climate urban design, Climate sciences, Environmental sciences

## Abstract

While outdoor thermal comfort (OTC) research in cold climate cities is expanding, comprehensive studies quantifying the seasonal impacts of urban morphology remain limited. This study fills a critical gap by establishing season-specific neutral Physiological Equivalent Temperature (PET) thresholds in Shenyang (Dwa climate, Northeast China) through 1,009 paired microclimate measurements and subjective surveys. Results show markedly lower thermal neutral points—12.5 °C in winter and 22.5 °C in summer—reflecting distinct cold-adaptive behaviors. This study provides empirical evidence quantifying the seasonal influences of urban morphology on OTC in a severe cold zone. The proposed Sky View Factor (SVF)-sensitive comfort framework advances climate-responsive design, providing empirical thresholds and parametric tools for cold-region planning. Findings contribute new evidence supporting seasonal adaptation theory and empirically offer a transferable methodology for similar high-latitude cities globally.

## Introduction

As cities continue to grow, the urban environment is undergoing profound transformations. Urban thermal environments are worsening due to global warming and urban heat island effects, both of which have direct impacts on public health, well-being, and outdoor thermal comfort (OTC)^1,2^. With increasing urbanization and the intensification of the UHI phenomenon, concern about OTC and its direct impact on residents’ quality of life is growing^3,4^.​ Optimizing OTC is thus essential for promoting social sustainability and urban vitality, particularly in the context of accelerating climate change^5^. Achieving Sustainable Development Goal 13 (SDG 13) requires urgent action to mitigate climate-related risks and improve the resilience of urban outdoor environments^6^.

Urban densification further alters surface energy balance, intensifying thermal environment challenges such as higher energy consumption and increasing risks of heat-related illnesses^7,8^. Urbanization may worsen OTC in winter by increasing hypothermia risks and heating demand, while summer effects differ. These issues are particularly pronounced in cold climate regions, where extreme seasonal contrasts and harsh winter conditions significantly exacerbate thermal discomfort. However, research on outdoor thermal comfort in such regions remains relatively scarce compared to studies conducted in temperate and hot climates. This gap is particularly salient given that a global review found more than half of thermal perception studies are focused on temperate climates, especially within China, leaving colder climates under-represented^9^.​

Thermal comfort studies commonly adopt the neutral Physiological Equivalent Temperature (PET) and the Universal Thermal Climate Index (UTCI) as core evaluation indicators, both of which are strongly climate-dependent. Early research primarily focused on temperate and humid tropical regions^10^. Bröde et al.^11^ studied UTCI in a Brazilian city, while Höppe^12^ focused on PET in Germany. In tropical rainforest climates (Af), neutral PET values tend to be higher. For instance, in Singapore, neutral PET ranges from 24.0 to 30.0 °C^13^. Similar trends are observed in tropical savanna climates (Aw), with studies in Brazil and Tanzania reporting values between 19.7 and 31.5 °C, accompanied by relatively wide acceptable comfort ranges^14–16^. In arid hot climates (BWh/BWk), including Israel, Tehran, and Cairo, residents showed strong heat adaptation with high neutral PET values (e.g., 25.1 °C in Tehran^17^, and large comfort ranges (e.g., 19.1–38.1 °C in Tempe, Arizona^18^;. In contrast, Mediterranean climates (Csa), such as Athens, Rome, and Tel Aviv, tend to present milder neutral thresholds, with PET values around 26.9 °C in Rome^19^ and UTCI values around 20.3 °C in Athens^20^. Similarly, oceanic climates (Cfb) such as Kassel, Szeged, Melbourne, and Glasgow generally exhibit lower neutral PET thresholds, in some cases as low as 9.0 °C in Glasgow^21^, along with relatively low UTCI neutral temperatures (e.g., 19.3 °C in Melbourne^22^;. Globally, tropical moist climate zones report the highest neutral temperatures, while snow climate zones have the lowest, with the latter also exhibiting the greatest seasonal variation in neutral temperature^9^, underscoring the critical need for season-specific studies in cold regions.

In temperate continental climates (Dwa), such as Beijing, Harbin, Dalian, Xi’an, and Tianjin, existing studies have revealed significant seasonal variations and regional differences in thermal neutrality (→ Appendix 1). However, critical knowledge gaps persist in colder Dwa and Dwb regions. Empirical data on PET neutrality remain limited, with only a few isolated studies in Harbin^7,23^ and Dalian^24^. While some studies cover multiple seasons (e.g^7,23^. robust head-to-head winter-summer comparisons remain scarce. Furthermore, the broader field faces challenges such as misunderstanding of benchmark terms and inappropriate calculations that can introduce bias^9^, complicating cross-study comparisons. While outdoor thermal comfort (OTC) in cold climates has garnered increasing attention, most research relies on general benchmarks or single-season evaluations. A critical knowledge gap remains regarding the ‘seasonal inversion’ effects of urban morphology—specifically the Sky View Factor (SVF)—in Severe Cold climate zones. Unlike temperate regions, high-latitude cities like Shenyang face a dual challenge: requiring solar access in winter while needing heat protection in summer.

Although the influence of SVF on thermal conditions is well-recognized^25^, its opposing seasonal impacts in cold climates are poorly quantified. Previous studies in hot-arid^26^, and subtropical climates^27^ have demonstrated that while low SVF (shading) improves summer comfort, it exacerbates winter discomfort by blocking desirable solar radiation. However, empirical evidence quantifying this specific trade-off in severe cold regions—particularly through continuous, synchronous measurements—remains limited. This highlights the urgent need to investigate urban form interactions, including geometry, vegetation, and materials, to optimize seasonal thermal adaptation in cold-region cities^4^.​

In addition to data gaps, factors such as urban form and behavioral adaptation also constitute limitations. However, what is particularly prominent is that the lack of key data has seriously hindered the development of climate-adaptive residential environments in the Dwa climate zone and the formulation of unified and effective standards (→ Appendix 2). One key variable, the Sky View Factor (SVF), has been recognized as a morphological indicator that modulates radiant heat exchange. SVF represents sky obstruction, modulating solar and infrared radiation fluxes^25^. Based on the results of^27^, we expect that afternoon temperature will tend to increase with SVF in both summer and winter, leading to degraded summer OTC (hyperthermia) with high SVF and degraded winter OTC (hypothermia) with low SVF. Moreover, current OTC benchmarks rarely reflect season-specific neutral PET values under real-life exposure scenarios. These gaps limit the development of resilient, behaviorally attuned outdoor spaces in rapidly urbanizing cold cities. Addressing these gaps is essential for proposing scientific planning and construction strategies to improve thermal comfort^4^ and could benefit from frameworks designed for systematic large-scale OTC exploration^3^.​

Building upon the methodological framework of^28^, this study responds to this research vacuum by focusing on Shenyang, which is the most populous city in Northeast China with over 8 million inhabitants, and has a continental Dwa climate (Koppen classification) with an annual temperature range exceeding 40 °C. Shenyang’s severe cold climate remains under-characterized in OTC literature, particularly regarding seasonal SVF effects. Our study addresses this by integrating joint PET-UTCI analyses with SVF bins under measured conditions, providing novel insights into adaptive design. Building on 1,009 field surveys and microclimate recordings, we aim to:

(1) Quantify neutral PET and UTCI thresholds for both winter and summer;

(2) Explore how SVF seasonally modifies thermal comfort across seasons;

(3) Propose a seasonally adaptive comfort model to inform urban design in severe cold climates.

By bridging climatological metrics, morphological variables, and behavioral adaptation, this study contributes to more nuanced thermal comfort frameworks and advances climate-adaptive spatial planning in under-studied high-latitude urban contexts. By providing seasonally differentiated, field-based OTC benchmarks, this study contributes to the understanding of Dwa climate zones, employing a novel bi-directional SVF model to quantify the effects of urban morphology.

## Methods

### Study area

**Fig. 1:**
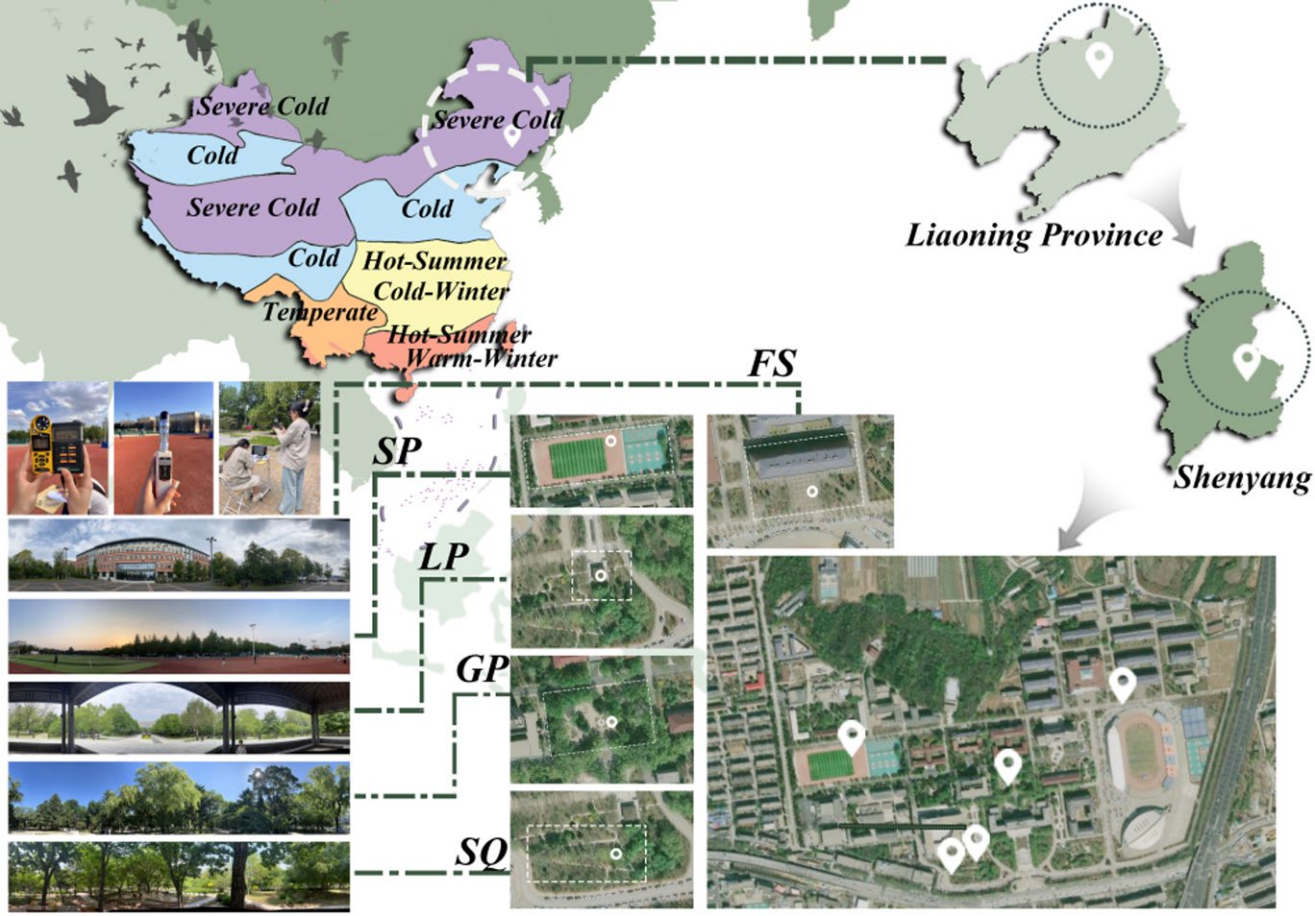
Location map of the five survey sites in Shenyang, China (Severe Cold Zone). The satellite imagery in Fig. 1 was obtained from Google Earth (Map data ^©^2024 Google). The map was generated using ArcGIS Pro version 3.0 (https://www.esri.com/en-us/arcgis/products/arcgis-pro/overview).

Shenyang is situated in Northeast China (41°11′–43°02′ N, 122°25′–123°48′ E). According to the Code for Thermal Design of Civil Buildings^29^, it is classified as a Severe Cold Zone C (→ Fig. 1). For international comparison, this region corresponds to the Dwa (Cold-dry winter, Hot-summer) category under the Köppen-Geiger classification. The city features distinct seasonal contrasts, with a dry, harsh winter and a hot, humid summer, making it an ideal laboratory for studying extreme seasonal thermal adaptation.

Meteorological data representing the historical climatic background were obtained from the Meteorological Dataset for Thermal Environment Analysis of Buildings in China^30^. According to this dataset, the coldest month is January (mean Ta = − 11.0 °C), and the hottest is July (mean Ta = 24.9 °C). During our field surveys, the measured microclimatic parameters (→ Table 2) fell within these typical seasonal ranges, ensuring the representativeness of the selected observation periods.

Compared with Beijing (Cwa climate), Shenyang experiences winters that are 6.2 °C colder on average, with 28 additional days of severe cold. The annual mean relative humidity is slightly higher (63.1% vs. 62% in Beijing), and the frequency of calm winds is 13% higher.

### Field study design

#### Public space selection

Based on stratified sampling along an SVF gradient, measurements were conducted at identical geographic coordinates in both winter and summer. SVF values were derived from fisheye lens photographs and calculated using RayMan Pro. Fisheye photographs were taken during peak daylight hours (10:00–14:00) on clear-sky days in January and July 2024. The site characteristics were summarized in → Table 1.

**Table 1:** Description of 5 selected spaces.

Site code	Site type	Winter SVF	Summer SVF	Thermal environment characteristics	Winter fish-eye Photo	Summer fish-eye Photo fish-eye photo
SP	Sports plaza (fully open)	1.000	1.000	Unshaded hard pavement (cold wind accumulation in winter; strong radiation in summer)		
FS	Front square (deciduous trees)	0.962	0.846	Deciduous trees, LAI = 2.8 in summer, light transmittance 38%		
GP	Garden plaza (mixed vegetation)	0.814	0.716	Composite vegetation of trees, shrubs, and grasses		
SQ	Shaded square (mixed vegetation)	0.641	0.073	Composite vegetation of trees, shrubs, and grasses		
LP	Landscape pavilion (structure)	0.500	0.142	Permanent shading structure		

#### Microclimate measurements

To investigate the OTC conditions in Shenyang’s Dwa climate zone, a mobile synchronous monitoring method was adopted. As shown in Fig. 1, five outdoor spaces with distinct Sky View Factor (SVF) gradients were selected to represent typical urban forms, including a plaza, pocket park, corridor pavilion, shaded grove, and open square. Trained researchers carried portable devices and conducted circular measurements across preset stations. Each point was monitored for 30 min to capture stable microclimatic conditions, with a full cycle of 2.5 h ensuring coverage of all sites within a consistent time window.

All instruments were fixed at a height of 1.5 m above ground level to simulate the average human perception height. Microclimatic parameters were recorded in real-time and were summarized in → Table 2.

**Table 2:** Instrument information.

Parameter	Instrument Model	Height	Accuracy	Range
Air Temperature(Ta)	Kestrel 4500	1.5 m	± 0.5℃	−40 to + 70 °C
Relative Humidity(RH)	Kestrel 4500	1.5 m	± 3%	0 to 100%
Wind Speed(V)	Kestrel 4500	1.5 m	± 0.1 m/s	0 to 40 m/s
Solar Radiation(G)	TES 1333	1.5 m	± 5%	0 to 2000 W/m²
Globe Temperature(Tg)	WBGT-213B	1.5 m	± 2℃	−5 to + 80 °C

#### Questionnaire survey

The questionnaire consisted of three sections. The first section focused on personal information, including gender, age, height, and weight^31^. Duration of residence in the city was also included and categorized into three groups for data analysis: less than 1 year, 1–4 years, and more than 4 years.

The second section addressed personal characteristics, including clothing insulation and the main activity status in the last 15 min^28^. These factors were used to reflect short-term thermal exposure prior to the survey.

**Fig. 2:**
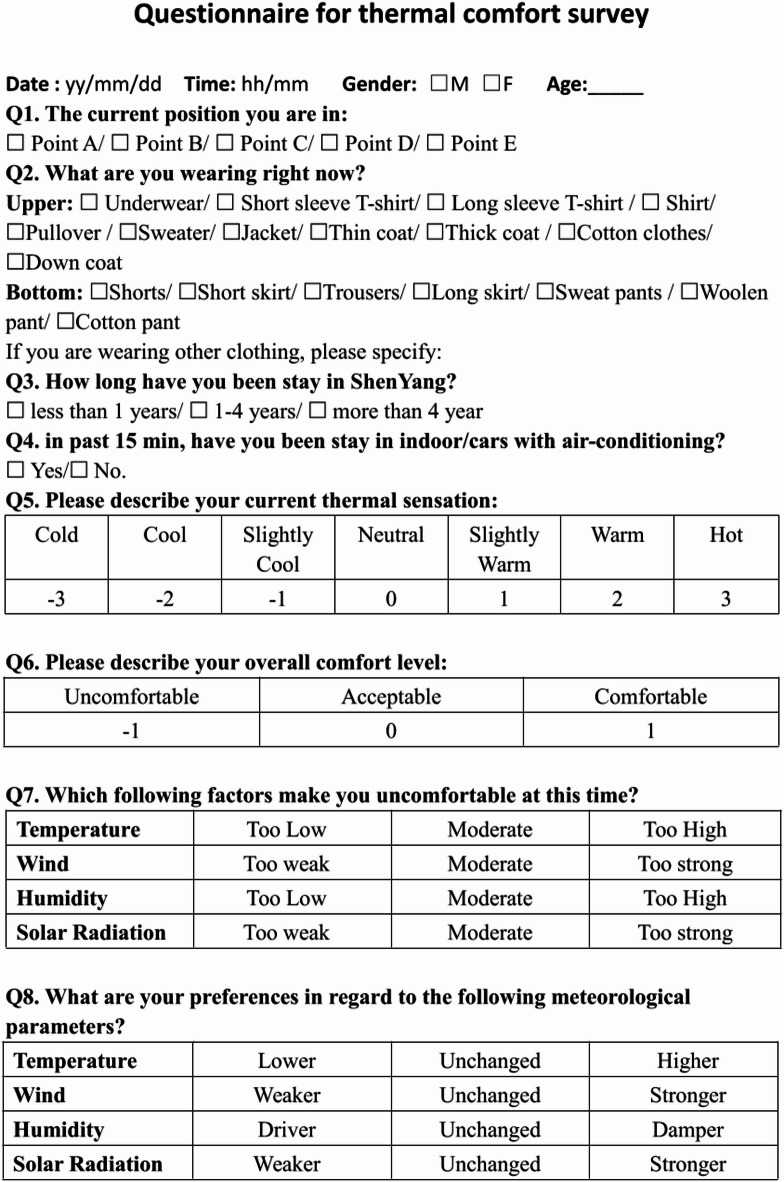
Workflow of synchronous microclimate monitoring and questionnaire administration.

The third section investigated thermal perception through three indices: thermal sensation vote (TSV), thermal comfort vote (TCV), and thermal preference vote (TPV). TSV referred to the subjective thermal sensation as reported by participants^32^, and followed the 7-point ASHRAE scale ranging from cold (− 3) to hot (+ 3)^33^. TCV assessed the perceived comfort level using a 3-point scale (uncomfortable, acceptable, comfortable), while TPV captured respondents’ desired thermal state based on the 3-point McIntyre scale. The survey was conducted in Chinese to target local residents, with translators available for non-Mandarin speakers. The detailed structure of these subjective thermal evaluation indices is illustrated in → Fig. 2.

#### Ethics approval

All methods were performed in accordance with the relevant guidelines and regulations. All experimental protocols were approved by the Ethic Committee of Human Studies, College of Forestry, Shenyang Agricultural University (Approval No. CF-EC-2025-233). Informed consent was obtained from all subjects and/or their legal guardian(s) prior to their participation in the survey.

#### Survey schedule

Field measurements were conducted across two distinct seasons, with detailed scheduling aligned with the Excel observation log. The observation periods, daily time frames, sample sizes, and weather conditions are summarized in → Table 3. Sample screening involved initial collection of 1,200 responses, with 1,009 valid after excluding incomplete or inconsistent entries. Exclusion criteria included missing demographic data or outlier thermal votes. Surveys were conducted daily during the indicated periods, including holidays, to ensure temporal representativeness.

**Table 3:** Date, times and weather conditions of field survey.

Season	Date range	Hours	Valid samples
Winter	09 Dec 2024–04 Jan 2025	08:00–17:30	456
Summer	18 May 2025–11 Jul 2025	08:00–18:00	553

### Thermal index calculation

PET was calculated using the RayMan Pro 3.1 software, a microscale radiation model developed by the Chair of Meteorology and Climatology at Albert Ludwigs University of Freiburg. The model is based on the original RayMan model developed by Matzarakis et al.^34^. Tmrt and PET outputs were generated from input variables including air temperature, relative humidity, wind speed, solar radiation, SVF, personal clothing and activity parameters. This software has been widely applied in OTC studies^35–37^. The input parameters included meteorological data (Ta, RH, Va, Tmrt), personal data (age, height, weight, clothing, and activity), and geographical data (longitude, latitude, and altitude).

UTCI was computed using the Pythermalcomfort package, an Python library developed by Tartarini at the University of California, Berkeley^32^. The inputs to this package were Ta, RH, Va, and Tmrt. Notably, Tmrt was calculated according to Eq. (1) in ISO 7726^38^. Tg (globe temperature, °C), Ta (air temperature, °C), and Va (wind speed, m/s) were obtained from field measurements. In this study, the globe emissivity (ε) was assumed to be 0.95 (black), and the globe diameter (D) was set to 150 mm. For UTCI calculations, wind speed at 10 m above ground (${v_{10}}$) was required and was corrected from measured wind speed at 1.5 m (${v_{1.5}}$) using the standard power-law wind profile (→ Eq. 2). The mean wind speed exponent (α) was taken as 0.33 for urban centers^39,40^.

1 $${T_{mrt}}={\left[ {{{({T_g}+273)}^4}+\frac{{1.10 \times {{10}^8}V_{a}^{{0.6}}}}{{\varepsilon {D^{0.4}}}}({T_g} - {T_a})} \right]^{1/4}} - 273.15$$

2 $${v_{10}}={v_{1.5}}{\left( {\frac{{{H_{10m}}}}{{{H_{1.5m}}}}} \right)^a}$$

where ${v_{10}}$ is the wind speed at 10 m above ground level (m/s), ${v_{1.5}}$ is the meteorological wind speed measured at 1.5 m (m/s), and α is the wind shear exponent. An α value of 0.33 was adopted to represent the aerodynamic roughness of the dense urban center (dense city center) in Shenyang.

After calculating the thermal comfort indices, three approaches were applied to determine the thermal benchmarks: linear regression, quadratic regression, and Probit analysis.

### Statistical analysis

**Fig. 3:**
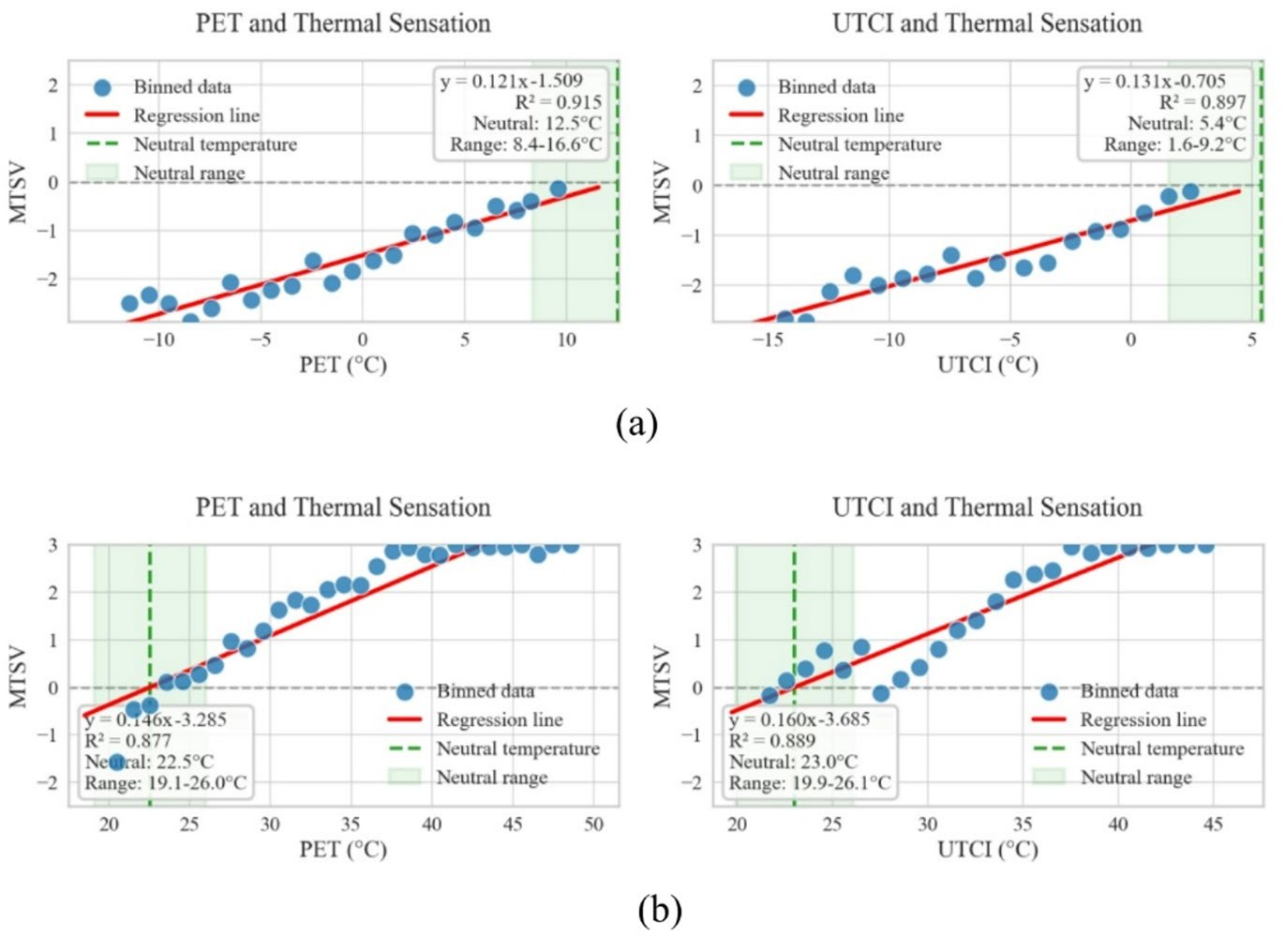
Regression analysis of average thermal sensation vote (MTSV) with PET and UTC: Winter (**a**) and Summer (**b**).

To minimize the scatter inherent in individual subjective responses and accurately derive thermal benchmarks, the “Mean Thermal Sensation Vote (MTSV)” method was applied. Individual TSV responses were aggregated into 1.0 °C intervals (bins) based on the corresponding PET/UTCI values. The mean TSV was calculated for each bin to generate the regression models (→ Fig. 3).

The Neutral Temperature (NPET/NUTCI) was determined by setting MTSV = 0 in the fitted linear equation ($y=ax+b$). The Neutral Range (NPETR) was defined as the temperature interval corresponding to an MTSV between − 0.5 and + 0.5^42,43^, calculated as(→ Eq. 3):

3 $$Rang{e_{lower}}=\frac{{ - 0.5 - b}}{a},\quad Rang{e_{upper}}=\frac{{0.5 - b}}{a}$$

## Results

### Thermal index distributions and driving factors

#### Normalized metrics: distributions and analytical methods

By applying the 1.0 °C binning method described in Sect. 2.4, the relationship between PET and MTSV was analyzed (→ Fig. 3). This approach effectively reduced individual vote scatter, though it resulted in fewer plotted points in the transitional neutral ranges due to the rapid seasonal shifts characteristic of the Dwa climate. Based on the regression equations shown in Fig. 3, the neutral PET ranges were calculated by substituting MTSV = ± 0.5. Linear regression demonstrated a strong positive correlation during summer conditions for both PET (slope = 0.1457, *p* < 0.001, 95% CI [0.125, 0.166], R² = 0.877) and UTCI (slope = 0.1603, *p* < 0.001, 95% CI [0.137, 0.184], R² = 0.889), with narrow neutral ranges indicating consistent thermal adaptation. In contrast, winter conditions showed significant and strong correlations, with PET (slope = 0.1208, *p* < 0.001, 95% CI [0.105, 0.137], R² = 0.915) and UTCI (slope = 0.1313, *p* < 0.001, 95% CI [0.110, 0.153], R² = 0.897). Despite the high correlation strength, the regression lines exhibited flatter slopes compared to summer, reflecting reduced thermal sensitivity and increased variability in cold-season thermal perception. These differential patterns highlight fundamentally distinct thermal adaptation mechanisms between seasons, with summer demonstrating stronger predictive relationships and winter showing adaptive broadening of thermal acceptance.

For Shenyang overall, summer NPET was 22.54 °C (PET) and 22.99 °C (UTCI), while winter NPET was 12.49 °C (PET) and 5.37 °C (UTCI). Group analysis by residential duration showed that summer NPET (PET) increased from 20.56 °C (< 1 year) to 24.37 °C (> 4 years), while winter NPET decreased from 13.83 °C to 9.61 °C across the same groups. A similar pattern was observed for UTCI, with summer neutrality rising from 24.92 °C to 26.11 °C, and winter neutrality declining from 11.48 °C to 3.50 °C.

The overall neutral PET ranges were 19.11–25.97 °C in summer and 8.35–16.63 °C in winter, as summarized in → Table 4. These results highlight clear seasonal differences and indicate that longer residential duration corresponds to higher heat tolerance in summer and stronger cold adaptation in winter.

**Table 4:** Season-specific neutral thresholds and acceptable ranges for PET and UTCI in Shenyang’s Dwa climate zone.

Season	Neutral PET (°C)	Acceptable range (°C)	Neutral UTCI (°C)	Acceptable range (°C)	Sample Ssize
Winter	12.5	8.4–16.6	23.0	19.9–26.1	456
Summer	22.5	19.1–26.0	5.4	1.6–9.2	553

#### Effects of sky view factor (SVF) on PET/UTCI distribution

**Fig. 4:**
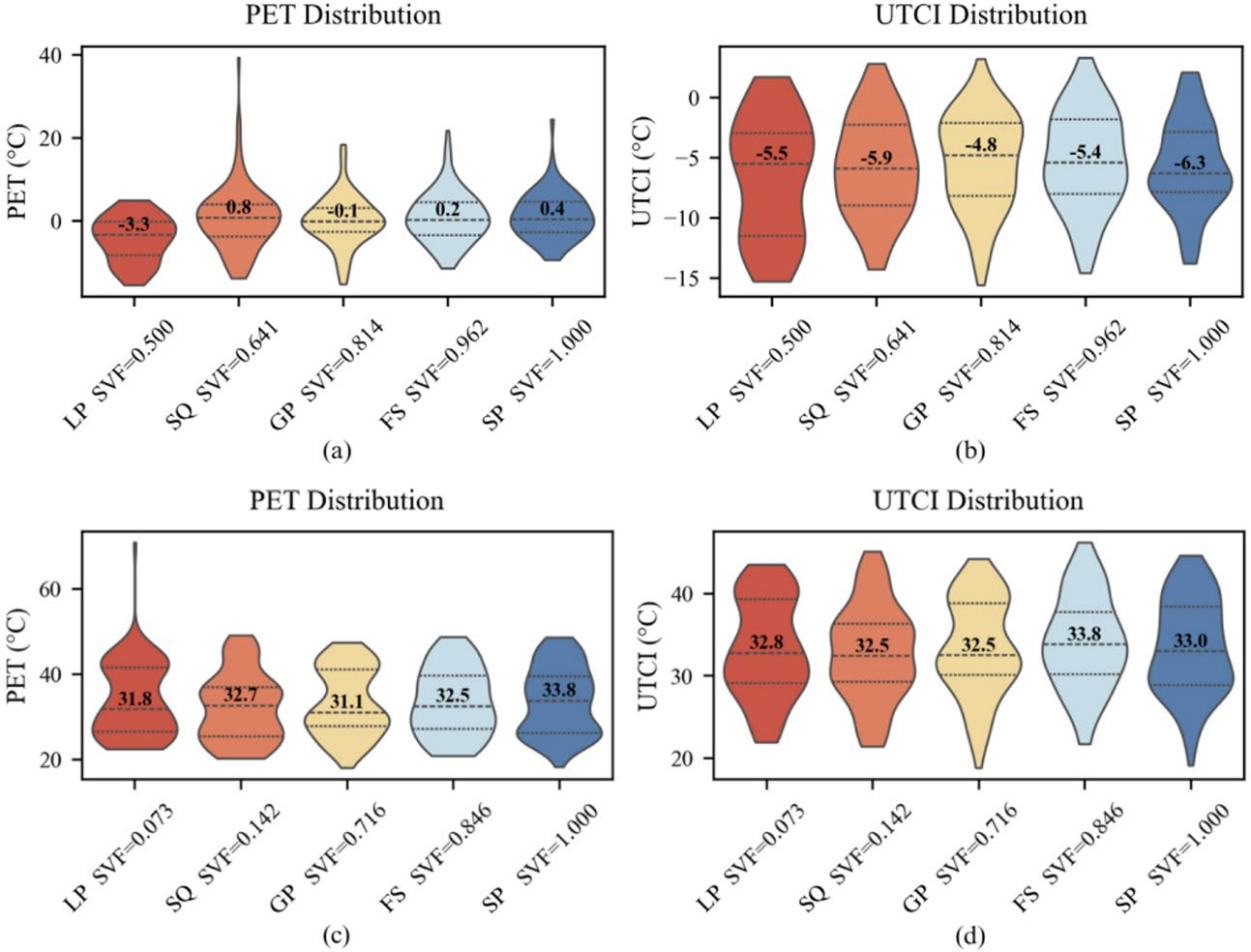
SVF-driven seasonal distributions of PET and UTCI: violin plots for winter (a, b) and summer (c, d) (*n* = 5).

Under both cold and hot conditions, sky view factor (SVF) systematically reshaped the distribution patterns of PET and UTCI (→ Fig. 4). In cold scenarios, when SVF = 0.500, the median PET was approximately − 3.3 °C, with an interquartile range from − 10 °C to 0 °C. As SVF increased to 1.000, PET values clustered around 0.4 °C, and the upper bound extended to 5 °C. Similarly, UTCI shifted rightward, from − 10 °C to − 5 °C. While SVF influenced PET distributions, the effects varied non-linearly across levels, suggesting complex interactions rather than simple monotonic trends. Summer UTCI distributions broadened from SVF = 0.073 to 0.142, with increased tail extremes.

In hot scenarios, the lowest SVF (0.073) produced the highest thermal load, with PET and UTCI medians reaching 31 °C and 32 °C, respectively, and long upper tails (20–45 °C) indicating elevated heat stress risks. At SVF = 0.142, both indices rose slightly (median ≈ 32 °C) while the distribution narrowed. With SVF = 0.846, median PET and UTCI peaked at 32.5–33.8 °C. At SVF = 1.000, the PET decreased slightly. In contrast, the UTCI values clustered near 33 °C. The distributions of both indices broadened compared to those at SVF = 0.846, indicating increased thermal environmental variability. At SVF = 0.641, median PET in cold conditions showed a sharp peak (+ 0.8 °C), while at SVF = 1.000, winter UTCI reached its lowest median (–6.3 °C) with a contracted interquartile range (→ Fig. 4).

Overall, SVF altered both central tendency and dispersion. PET and UTCI medians fluctuated across SVF levels without a monotonic trend, highlighting complex interactions. While some patterns emerged, trends were not robust across all SVF levels. These findings confirm the important role of SVF in regulating radiation loads and provide a basis for micro-scale thermal environment design.

#### Seasonal comparison characteristics of PET/UTCI

**Fig. 5:**
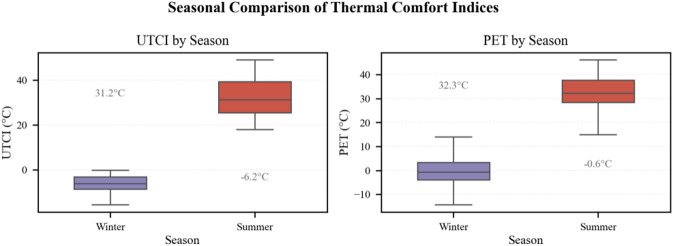
Seasonal contrast of median PET and UTCI: boxplots for winter and summer.

PET and UTCI were positively correlated throughout the year, but SVF modulated the strength of their association. In winter, correlation coefficients declined from *r* = 0.70 (SVF = 0.500) to *r* = 0.55 (SVF = 1.000); in summer, from *r* = 0.79 (SVF = 0.073) to *r* = 0.66 (SVF = 0.716). Winter UTCI had a median of − 5 °C (mean − 6.2 °C) and PET 0 °C (mean − 0.6 °C), while summer UTCI and PET both had medians of 30 °C, with means of 31.2 °C and 32.3 °C respectively, illustrating the contrast between narrow cold stress and wide heat stress (→ Fig. 5).

**Fig. 6:**
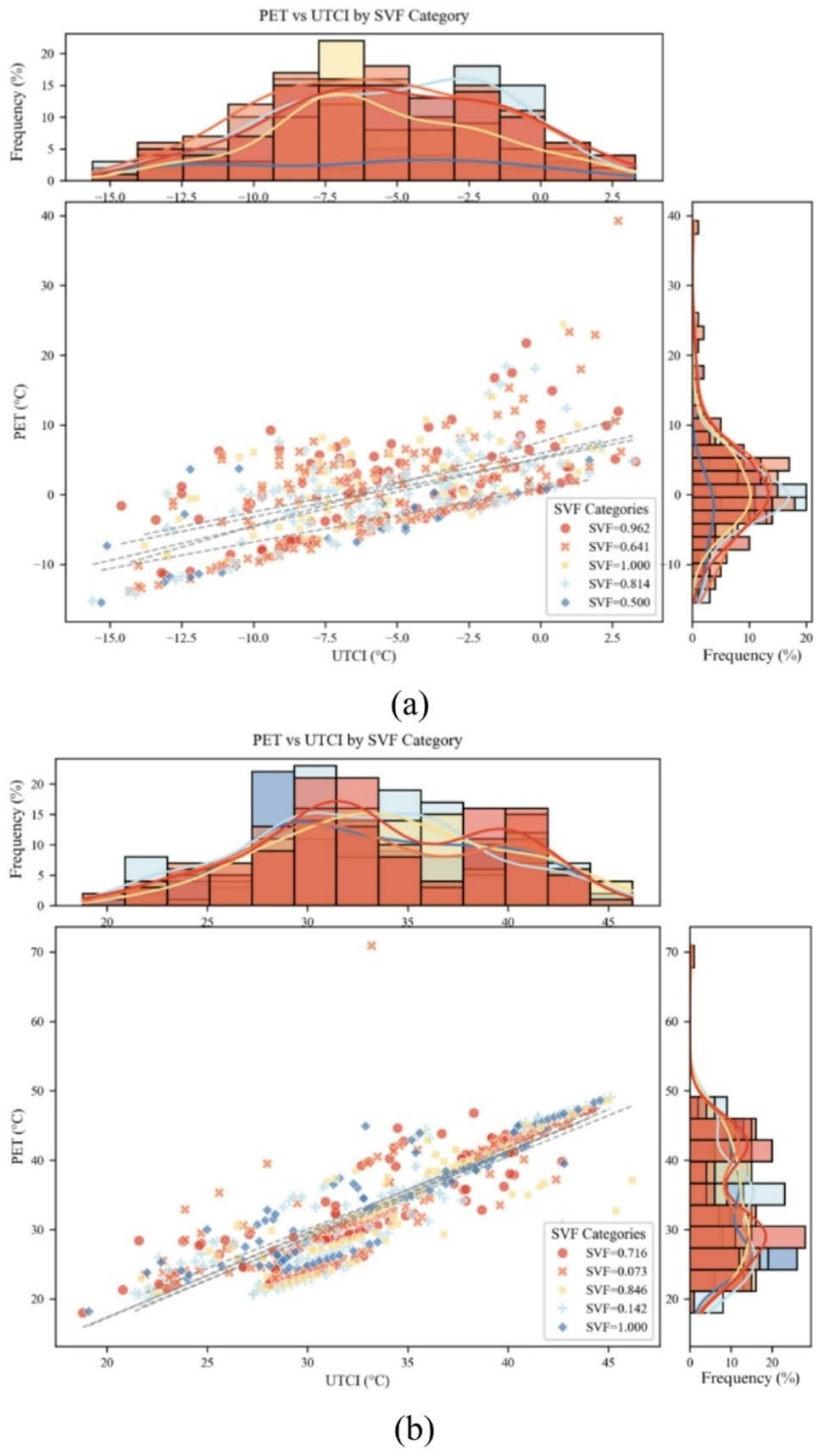
PET-UTCI scatter colored by SVF revealing convergent-divergent shift across sky-openness: Winter (**a**) and Summer (**b**).

The joint distribution of PET and UTCI was analyzed across multiple SVF categories to examine their seasonal coupling patterns. In summer, a strong positive correlation was observed between PET and UTCI across SVF values of across all SVF levels. Most data points clustered along a nearly linear band (UTCI: 20–45 °C; PET: 20–50 °C), with marginal density plots revealing distributional differences by SVF. For example, SVF = 0.716 showed a sharper, more concentrated peak, indicating a higher frequency of specific combinations of PET and UTCI. These results suggest that SVF modulates not only absolute thermal levels but also the frequency of particular thermal states. In winter, PET and UTCI also showed a positive association across SVF values of across all SVF levels (→ Fig. 6). Data ranged more widely (UTCI: −20 to 10 °C; PET: −20 to 40 °C), but clustering patterns varied by SVF. Some categories showed tighter distributions around mid-range values, suggesting SVF influences thermal consistency in cold conditions.

### Subjective heat perception and evaluation characteristics

#### Perception distribution and microclimate weighting

**Fig. 7:**
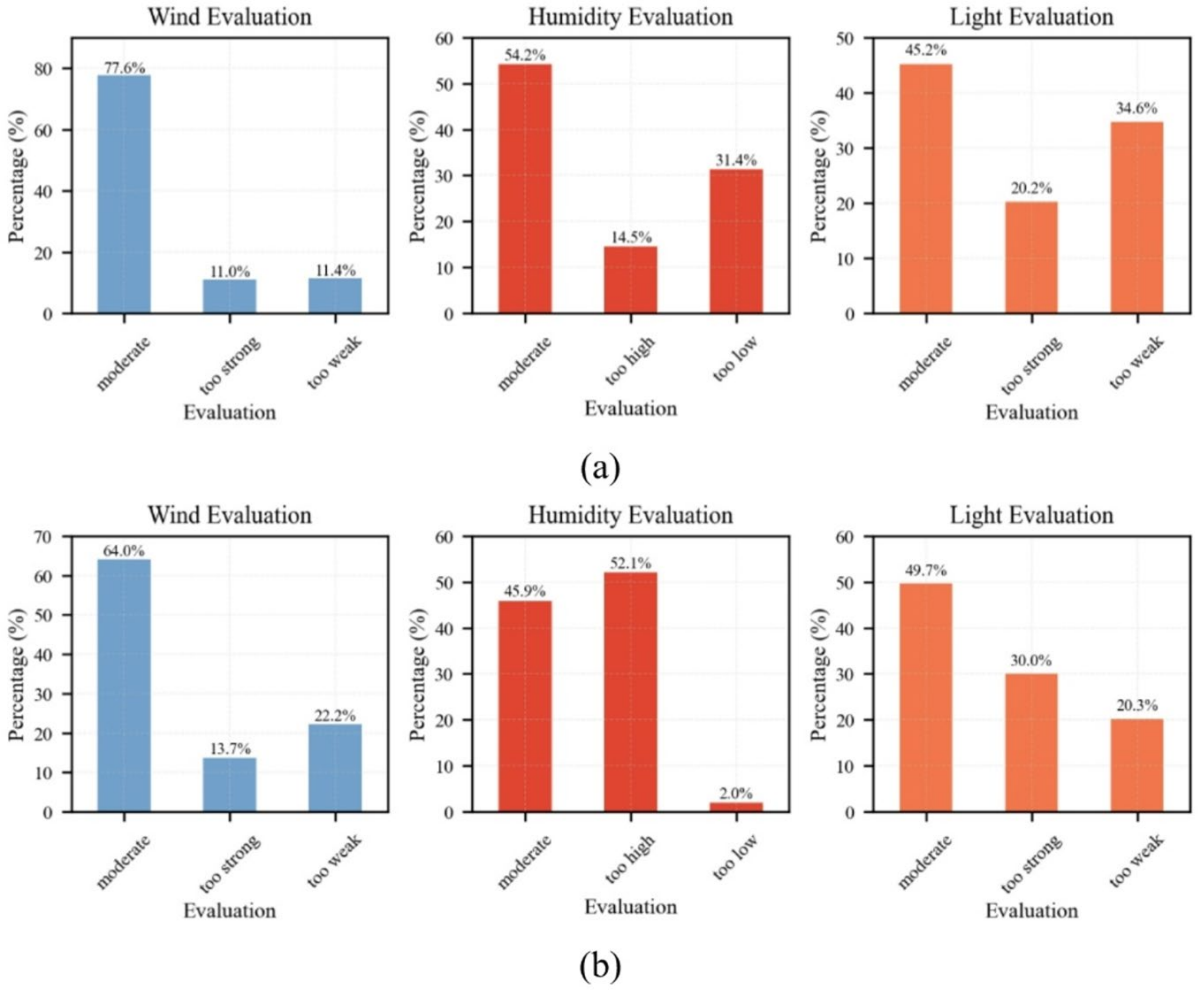
Evaluation results of each meteorological variable: Winter (**a**) and Summer (**b**). Despite similar distributions of perception, the sources of discomfort varied: in winter, “too low RH” emerged as the dominant discomfort factor due to low humidity exposure during the cold season, whereas in summer, high temperatures and strong solar radiation elevated the negative influence of both “too strong light” and “too low RH” on thermal comfort. These seasonal shifts indicate that the perceived importance of each microclimatic factor is context-dependent.

Building upon previous findings that thermal sensation votes (TSV) are primarily influenced by microclimatic variables such as globe temperature (TG), air temperature (TA), relative humidity (RH), and wind speed (VA)^43,44^, this study further examines the subjective perception of wind speed, humidity, and solar radiation, as well as their respective impacts on outdoor thermal comfort (OTC) in Shenyang during winter and summer. As shown in → Fig. 7 responses from 456 participants in winter and 533 in summer revealed different distributions across seasons: In winter, 77.6% considered wind speed “moderate,” 11.0% “too strong,” and 11.4% “too weak”; 54.2% perceived RH as “moderate,” 14.5% as “too high,” and 31.4% as “too low”; and 45.2% rated light intensity as “moderate,” 20.2% as “too strong,” and 34.6% as “too weak.” In summer, 64% considered wind speed “moderate,” 13.7% “too strong,” and 22.2% “too weak”; 45.9% perceived RH as “moderate,” 52.1% as “too high,” and 2% as “too low”; and 49.7% rated light intensity as “moderate,” 30% as “too strong,” and 20.3% as “too weak.”

#### Analysis of the relationship between TSV and TCV

**Fig. 8:**
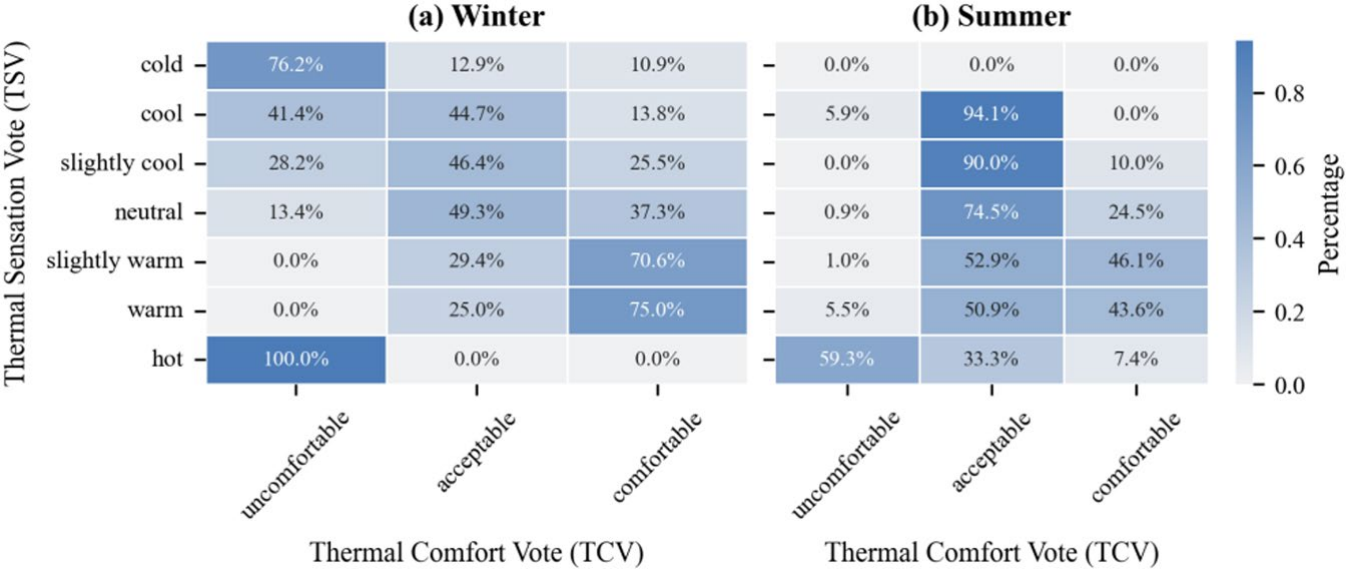
Thermal comfort vote (TCV) distribution across 7-point thermal sensation votes (TSV).

As shown in Fig. 8 (a) and (b), the relationship between thermal sensation and comfort votes exhibited distinct seasonal patterns.

In winter, the highest proportion of ‘Comfortable’ votes was not observed at the “Neutral” sensation (TSV = 0), but rather at “Slightly Warm” (TSV = + 1). Specifically, when participants felt “Slightly Warm,” 70.6% rated the environment as comfortable, significantly higher than the 37.3% observed under “Neutral” conditions. This indicates a psychological preference for warmth (thermal alliesthesia) in severe cold environments.

In summer, the trend shifted. The highest acceptability appeared at the ‘Cool’ and “Slightly Cool” levels (reaching 94.1% and 90.0% respectively), confirming that residents prioritize heat relief. These results correct the limitation of aggregated annual analysis and highlight that ‘thermal neutrality’ does not always equate to “optimal thermal comfort” in extreme climates.

These results demonstrate a clear association between thermal sensation and comfort perception: the “neutral” sensation corresponds to the highest proportion of comfort, while both “cold” and “hot” sensations are strongly linked to discomfort. This pattern suggests that thermal sensation significantly influences how individuals evaluate the thermal acceptability and comfort of their environment.

### Parameter interrelations and functional mechanisms

#### Three-dimensional interaction among air temperature, relative humidity and PET

As shown in → Appendix 4, a 3D scatter plot was used to illustrate the relationship among air temperature (Ta), relative humidity (RH), and physiologically equivalent temperature (PET) for the winter and summer seasons, with sky view factor (SVF) represented by a continuous color gradient. In winter (Ta: − 15 to 5 °C; RH: 20–60%; PET: − 10 to 30 °C), most data points were clustered at low Ta, moderate RH, and near-neutral PET levels (around 0 °C). In summer (Ta: 17.5 to 37.5 °C; RH: 20 to 100%; PET: 20 to 70 °C), data were mainly concentrated within the Ta range of 25–30 °C, RH range of 50–80%, and PET of 20–50 °C.

#### Seasonal characteristics and SVF moderation effects

A total of 1,009 thermal comfort votes (TCV) were collected throughout the year. Among them, 45.3% were classified as “acceptable,” 30.8% as “uncomfortable,” and 23.9% as “comfortable,” suggesting that thermal acceptability was dominant, although discomfort remained considerable. Seasonal variation significantly influenced TCV distribution (χ² = 55.1, *p* < 0.001). In winter (*n* = 456), 43.0% of responses were marked as “uncomfortable,” with only 20.8% rated as “comfortable.” In contrast, summer votes (*n* = 553) showed improved thermal perception, with “acceptable” and “comfortable” proportions rising to 51.6% and 26.1%, respectively, while “uncomfortable” dropped to 22.3% (see → Appendix 5).

**Fig. 9:**
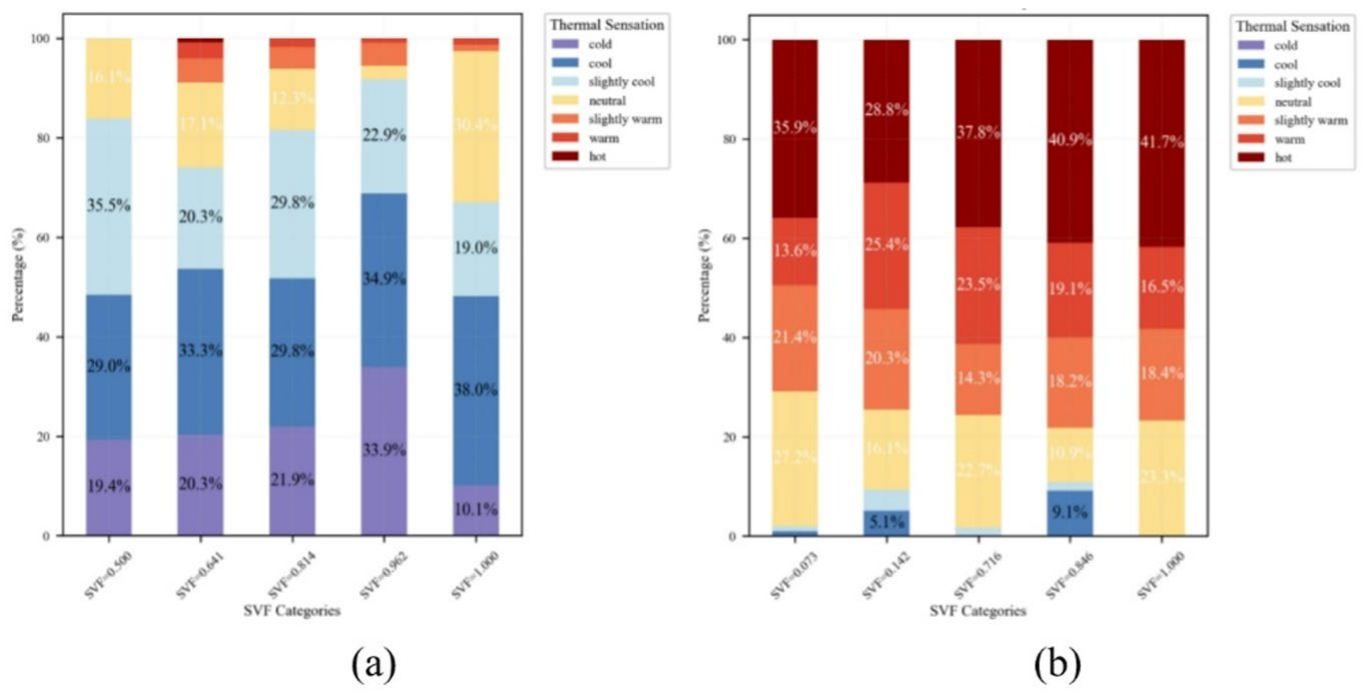
Seasonal TSV distributions across SVF categories: (**a**) winter and (**b**) summer.

As shown in → Fig. 9, the inclusion of sky view factor (SVF) further revealed that local openness significantly reshaped the distribution of thermal sensation votes (TSV), primarily by altering solar radiation exposure. In winter, the proportion of “cold” responses exhibited nonlinear fluctuations with increasing SVF, peaking at 33.9% when SVF = 0.962, then dropping sharply to 10.1% at full openness (SVF = 1.000), where “neutral” votes simultaneously increased to 30.4%. In summer, TSV mainly comprised “hot,” “warm,” “slightly warm,” and “neutral” across all SVF levels, but their relative proportions varied with SVF. For instance, “hot” votes rose from 35.9% at SVF = 0.073 to 41.7% at SVF = 1.000. The “cool” category was only observed at SVF = 0.142 and 0.846, with limited proportions of 5.1%–9.1%. While no global trend was observed across all five SVF levels, localized patterns emerged at levels 2–4.

### Temporal dynamics of thermal environments

#### Diurnal shifts of thermal indices with wind interaction

The summer thermal environment in Shenyang during 2025 exhibited high-amplitude fluctuations. The mean values of PET and UTCI were 33.5 °C and 33.2 °C, respectively, with their density curves overlapping in the “heat stress” range. However, PET extended further toward the higher temperature end. Between June and July, PET oscillated between 20 and 45 °C from 08:00 to 17:00, peaking at 14:00, while UTCI peaked one hour later at 15:00. Notably, 22% of PET readings between 12:00 and 14:00 exceeded 40 °C, indicating a concentrated period of extreme heat exposure. Female participants reported “hot” sensations 17% more frequently than males. When wind speed exceeded 2 m/s, PET decreased by an average of 3.2 °C, highlighting the cooling effect of wind (see → Appendix 6).

In contrast, winter presented a markedly different thermal profile. The average PET was − 1.2 °C, with values ranging from − 15 °C to 25 °C. PET and UTCI density curves diverged in the “comfortable/slightly cool” range, with distinct differences in peak positions. Between December 1 and 12, PET followed a diurnal pattern, with lower values in the morning and higher values in the afternoon. The daily temperature range is between 8 and 10 °C. Based on the trend in Fig. 17, the PET value is inferred to be below − 10 °C during 35% of the early morning periods (04:00–08:00), indicating significant cold exposure. Under wind speeds greater than 2 m/s, PET decreased by an additional 4.1 °C, demonstrating the compounding effect of wind chill. Minimum values typically occurred between 06:00 and 07:00 (see → Appendix 7).

#### Seasonal PET dynamics and SVF moderation in Shenyang

**Fig. 10:**
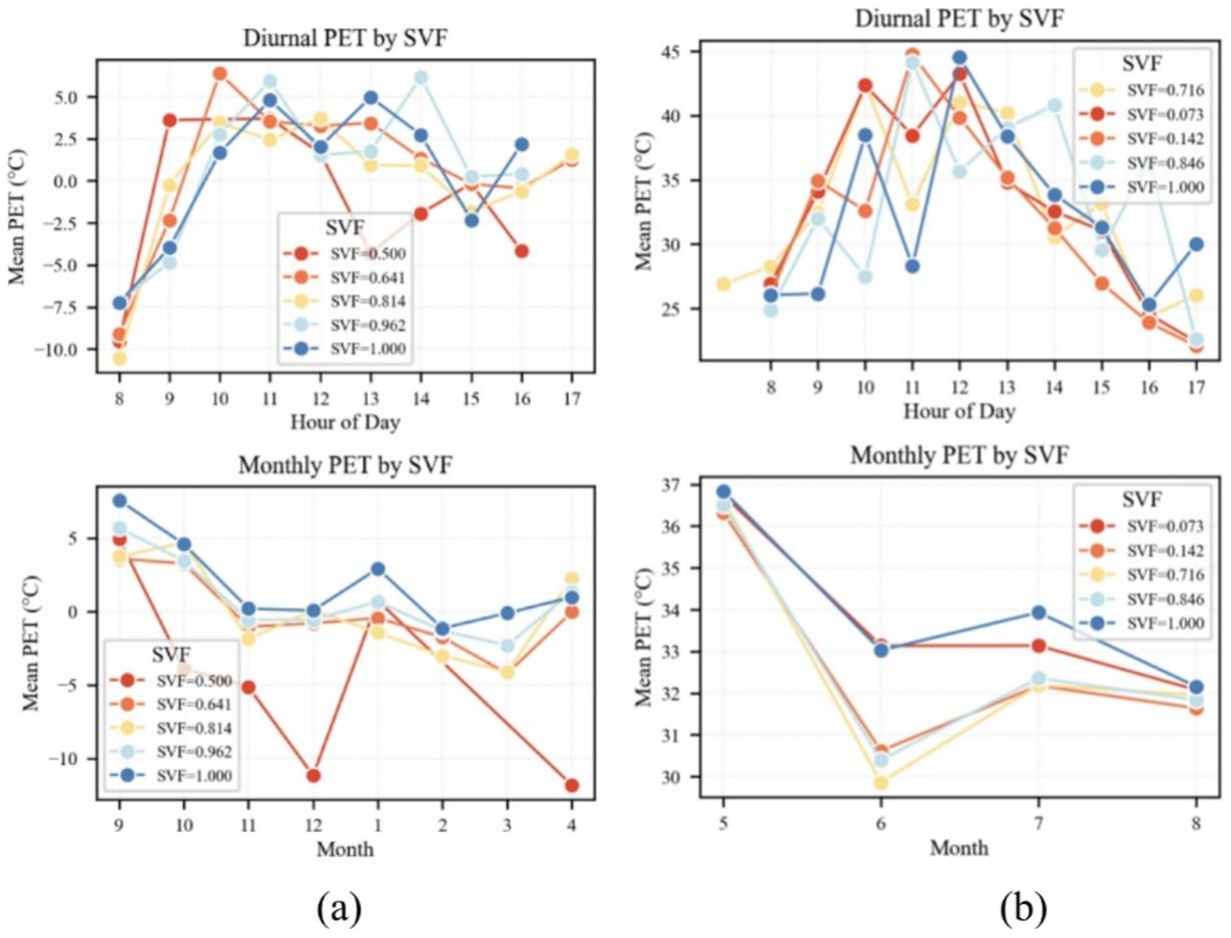
Diurnal and monthly PET trajectories across SVF classes: (**a**) winter and (**b**) summer.

As shown in → Fig. 10a, during winter days (08:00–17:00), PET exhibited pronounced fluctuations across different SVF categories. For SVF = 0.500, PET sharply increased from − 9 °C at 08:00 to 5 °C at 10:00, then declined to − 4 °C by 13:00. In contrast, under SVF = 1.000, PET rose from − 7 °C at 08:00 to 5 °C at 11:00, indicating that open spaces can elevate PET by 12 °C within a 3-hour window. This warming is primarily driven by solar radiation. On the monthly scale, PET under SVF = 0.500 decreased from 4 °C in September to − 11 °C in December and further to − 12 °C in April. Meanwhile, PET under SVF = 1.000 decreased from 7 °C to 0 °C in December and rebounded to 2 °C by April. Higher SVF values enable faster daytime heating due to lower thermal inertia, thus reducing the intensity and duration of winter thermal discomfort.

Summer PET dynamics were even more intense (→ Fig. 10b). At most SVF levels, PET rose rapidly from 08:00, peaking at 45 °C under SVF = 1.000 by 11:00, followed by a sharp decline to 25–30 °C after 15:00. All levels showed similar trends except SVF = 0.846. For each 0.1 decrease in SVF, the PET peak occurred approximately 20 min earlier, with a corresponding drop in peak temperature of 1.5–2.0 °C. While the trend is evident across most SVF levels, it is not consistent at all levels. On the monthly scale, PET typically reaches 37 °C in May. In June, increased cloud cover and rainfall reduced PET to 30–33 °C across most SVF groups, followed by a rebound and clustering during July and August. The PET drop was most pronounced under SVF = 0.073 (–4 °C) and least under SVF = 1.000 (–1.5 °C), indicating that higher levels of enclosure are associated with greater monthly variability in thermal conditions. Figure 10b shows similar patterns at most SVF levels, though these findings may not be generalizable to all levels.

## Discussion

**Fig. 11:**
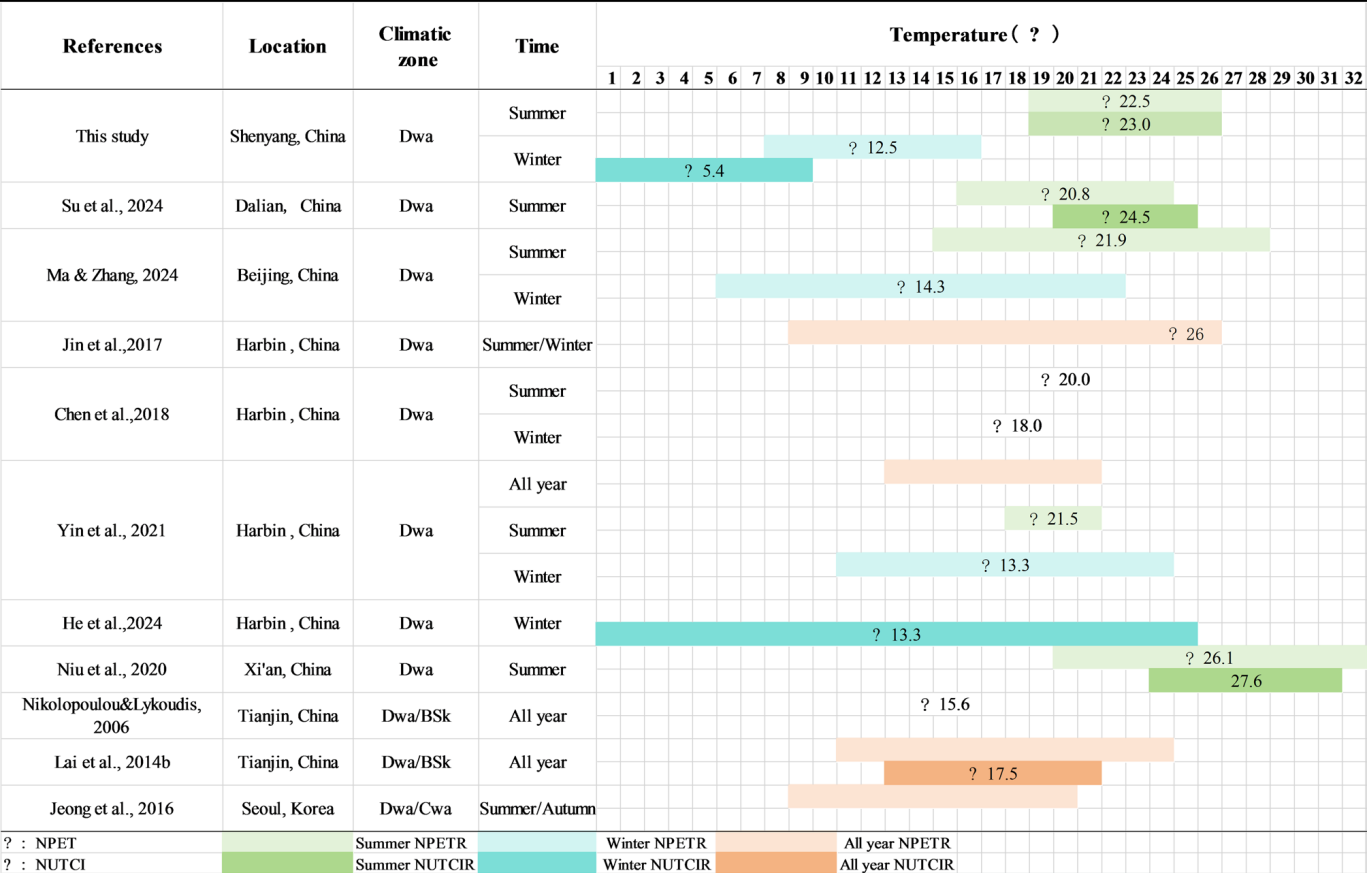
Comparing thermal benchmarks of researches in Dwa zone.

To determine the OTC of the Dwa zone, this study compared Shenyang’s results with those of other studies in the same climate. Thermal perception evaluations vary depending on the design requirements of different spaces and resident activities. For guiding outdoor thermal environment design, summer and winter are considered the typical seasons. Combining results from different seasons may differ from single-season findings^45–47^, potentially leading to a misrepresentation of the actual conditions in typical seasons. As illustrated in →Fig. 11, a comparative analysis within the same seasons is essential to mitigate this risk.

### Comparison with existing studies

This study establishes, for the first time, the neutral PET benchmarks for both summer and winter in Shenyang’s Dwa climate zone: 22.5 °C in summer and 12.5 °C in winter. This value in winter is considerably lower than the commonly reported summer neutral PET range of 18–23 °C in temperate oceanic climates^21,48^. The winter value is approximately 2–3 °C lower than the lower bounds reported for Umeå (14.4 °C^49^; and Warsaw (6.3–21.8 °C^50^;, which provides empirical support for the ‘cold adaptation hypothesis’ in outdoor thermal comfort research. This supports the cold adaptation hypothesis by showing that lower NPET values in Shenyang reflect physiological acclimatization to severe winters, as evidenced by longer residence duration effects.

Compared with previously published NPET data from Dwa regions, Shenyang’s summer value of 22.5 °C falls near those of Beijing (21.9 °C^28^;, Harbin (20.0–21.5 °C^7,23^, and Dalian (20.8 °C^24^, but is lower than the value reported for Xi’an (26.1 °C^24^. This suggests a latitudinal gradient of approximately − 0.9 °C per 1° increase in latitude within the same climatic zone (→ Fig. 11). The winter NPET of 12.5 °C is also lower than Harbin’s reported values (13.3–18.0 °C^7,23^; and Beijing (14.3 °C^28^;, further indicating enhanced cold adaptation in more severe environments. This anomaly may be due to Shenyang’s urban heat island effects or stronger cold adaptation among residents, despite its geographical position.

In terms of thermal sensitivity, the regression slope of PET-thermal sensation vote (PET-TSV) was found to be 0.146 in summer and 0.121 in winter, placing Shenyang between Dalian (0.13 in summer^24^; and Harbin (0.073 in summer– 0.09 in summer^7,51^. This implies that Shenyang residents exhibit lower thermal sensitivity than those in Dalian, but higher than those in Harbin’s longitudinal studies with stricter volunteer screening. These differences highlight the influence of cultural background, measurement periods, and site heterogeneity on thermal response slopes.

A similar pattern is observed for UTCI: the neutral UTCI in Shenyang was 5.4 °C in winter, significantly lower than Harbin’s 11.5–20.4 °C^51^, and 23.0 °C in summer, slightly lower than Dalian’s 24.5 °C. These results confirm the synchronous offset patterns across indices in severe cold zones, though UTCI tends to amplify wintertime differences due to its incorporation of wind chill effects.

Data from Tianjin (15.6 °C for PET, 17.5 °C for UTCI^45,47^; were excluded from direct comparison due to the inclusion of transitional season measurements. Nonetheless, its upper winter threshold partially overlaps with Shenyang’s winter acceptability limit of 16.6 °C, underscoring the importance of seasonal specificity in neutral temperature estimation.

Taken together, the results establish Shenyang as a distinctive reference point in the Dwa climate zone, characterized by a ‘moderate summer, very cold winter’ thermal preference pattern. These benchmarks provide valuable reference points for establishing unified outdoor thermal comfort standards in cities with cold climates. This study offers empirical validation for the cold-adaptation hypothesis in urban outdoor thermal comfort. In Shenyang, the observed neutral PET values was 12.5 °C in winter, both lower than those reported in other Dwa cities, indicating stronger physiological and behavioral adaptation among long-term residents. This adds to the understanding of regional acclimatization gradients, potentially governed by latitude and winter severity.

### Comparative analysis of neutral temperature ranges and thresholds

Using TSV = ± 0.5 as the threshold for acceptability, the NRPET in Shenyang was determined to be 8.4–16.6 °C in winter and 19.1–26.0 °C in summer. These ranges differ substantially from those reported in previous studies in the Dwa climate zone. For example, the NRPET for Tianjin was reported as 11–24 °C^46,51^, which includes winter data and thus shows a higher bottom threshold than Harbin’s 10.9–19.3 °C^51^. This supports the winter-dominant pattern by showing that mixed-season samples may overestimate comfort ranges, as seen in Tianjin’s data^46,47^. In Harbin,) defined the acceptable range as 9–26 °C by combining winter and summer neutral values, yielding a range width of 17 °C, substantially broader than Shenyang’s separate winter (8.2 °C) and summer (6.9 °C) ranges. This range is still 7 °C wider than the 10 °C range obtained using the same definition method in this experiment. This illustrates how non-seasonal grouping may overestimate thermal acceptability.

The NRPET in Dalian was reported as 16.9–24.6 °C^24^, with a nearly identical upper threshold to Shenyang’s summer value (26.0 °C), but a 2.8 °C higher lower threshold. This reflects Dalian’s warmer Cold Zone A thermal zone classification and reduced cold tolerance. In terms of UTCI, Dalian’s NRUTCI was 17.1–23.7 °C, representing a narrower range shifted toward higher temperatures compared to Harbin’s 10.6–20.6 °C^52^. This corresponds to a higher regression slope between UTCI and TSV in Dalian (0.13) than in Harbin (0.073), indicating stronger thermal sensitivity and consequently a narrower acceptable range. Shenyang’s slopes fall in between, with 0.121 in winter and 0.146 in summer, and the corresponding range widths also lie between the two cities (as shown in → Fig. 11^53,54^. These findings are consistent with those of Cohen et al.^55^(cited in^54^, who reported convergence in upper thresholds and divergence in lower ones across regions.

Further analysis was conducted to examine the influence of residence duration on thermal perception (see → Appendix 1). In both seasons, residence duration exerted a significant impact on thermal adaptation. In summer, residents who had lived in Shenyang for 1–4 years showed lower NPET and NUTCI values than those with over 4 years of residence, indicating higher sensitivity and lower thermal tolerance among short-term residents. In contrast, in winter, long-term residents had lower neutral index values than newer residents, suggesting better adaptation to cold.

These results highlight the need to consider residence duration when designing urban thermal environments. In newly developed or climate-transitioning areas, greater attention should be paid to addressing the comfort needs of short-term residents. For long-term residents, improvements in vegetation and building envelope performance may offer enhanced thermal comfort (see → Appendix 3).

Notably, the regression slopes between PET and TSV reveal varying thermal sensitivity across cities and seasons. Shenyang residents displayed moderate sensitivity (PET-TSV slope 0.121–0.146), suggesting a balance between heat tolerance and cold resilience. A balance may be achieved through low thermal sensitivity, enhancing adaptability. Compared to Harbin (lower slope, higher cold tolerance) and Dalian (higher slope, less adaptation), Shenyang represents a intermediary adaptive profile within the Dwa zone.

### Acceptable temperature range

Based on 1,009 field questionnaires and synchronous microclimate measurements, this study has recalibrated the acceptable thermal ranges in Shenyang under the 90% acceptability criterion (i.e., TCV ≥ 0). The ARPET ranged from − 10.5 to 10.4 °C in winter and from 20.1 to 45.4 °C in summer, while the ARUTCI ranged from − 11.2 to 9.7 °C and from 20.6 to 48.8 °C, respectively. Compared with Harbin’s summer ARPET of 11.6–35.9 °C^51^, Shenyang showed an higher upper threshold but a substantially lower bottom limit, extending by 8.5 °C. For UTCI, the difference is even more pronounced, with Shenyang’s upper limit exceeding Harbin’s by 13 °C due to stronger wind-chill effects. In winter, the contrast is greater: Shenyang’s PET lower threshold is 15 °C lower than Harbin’s estimate, highlighting the high cold tolerance of residents in severe cold regions.

In Dalian, the summer ARPET was 17.3–29.6 °C, significantly narrower and shifted toward the comfort zone compared to Shenyang. Although Dalian had a higher mean air temperature (28.6 °C vs. 27.7 °C in Harbin) and a larger daily temperature range (7.1 °C vs. 4.9 °C), the regression slope between PET and TSV in Dalian (0.13) was higher than that in Harbin (0.073–0.09), indicating stronger thermal sensitivity among residents, resulting in a narrower acceptable range. According to^19^, the difference between the two cities would be lower than the difference between multiple points in the same city. Dalian’s data were collected solely from three squares, while Harbin and Shenyang include a range of urban spaces (squares, streets, campuses), which may partly explain the wider AR range in the latter two cities (see → Fig. 11).

The relationship between neutral and acceptable thresholds was found to vary geographically. In Shenyang, the lower limit of acceptable PET was 12.9 °C below the neutral threshold, while for UTCI the gap was only 1.5 °C. In Dalian, both the acceptable PET and SET* lower limits were below their respective neutral thresholds, while UTCI showed an opposite shift, with the acceptable lower threshold exceeding the neutral one. This pattern, showing broader ranges in severe cold climates and compressed ranges in temperate coastal zones, supports the concept of a flexible thermal zone proposed by^41^, wherein behavioral and physiological adaptation enables a greater overlap between neutral and acceptable zones under extreme cold conditions, contrasting with mild environments. The seasonal separation of field data in Shenyang provides the first direct evidence from the Dwa climate region supporting this hypothesis, offering a more inclusive and seasonally refined thermal benchmark for cold-region urban design. Furthermore, our findings suggest that thermal acceptability ranges are more elastic in winter, likely due to lower metabolic stress and behavioral insulation. The observed overlap between neutral and acceptable ranges implies that cold-region residents possess a broader thermal tolerance band, a hypothesis that challenges traditional static comfort models and supports the concept of a “flexible comfort zone”^41^.

### Thermal stress categories

The OTC indices in this study were developed using both PET and UTCI models, with classifications based on 7-point TSV and 3-point TCV scales to ensure intuitive public interpretation of thermal stress levels^13,56^. When compared to the three Harbin studies, methodological discrepancies led to inconsistent neutral thresholds across studies. For instance, X. Chen et al.^7^ reported a winter NPET of 18 °C using discriminant analysis; however, this value fell within the ‘Slightly Cool’ category rather than the true neutral zone, highlighting how methodological variations can amplify outcome bias; this finding aligns with Kenawy & Elkadi^45^ methodological concerns regarding seasonal mixing.

In contrast, Nikolopoulou & Lykoudis^46^ carried out a Tianjin study covering all four seasons but with field measurements restricted to 10:00–16:00. Their definition of neutral PET at ± 0.5 TSV, combined with short sampling windows and seasonal mixing, resulted in further compression of the thermal comfort range. The present study improves upon these limitations by applying full-day rolling measurements (08:00–18:00) during both winter and summer, supplemented by a regression–probit combined model. This approach not only bridges data gaps but also mitigates potential time-bias distortions.

Regarding index amplitude, Shenyang’s PET-based thermal stress ranges were slightly narrower than Harbin’s^41^. Pantavou et al.^20^ demonstrated that UTCI’s dynamic clothing insulation decreases markedly at high temperatures, enhancing index sensitivity and consequently widening the acceptable thermal range. This wider UTCI band reflects a higher adaptive capacity among Shenyang residents in the context of cold climate conditions, contrasting sharply with the narrower thermal bands observed in Dalian^24^. These results reaffirm the critical importance of methodological standardization for establishing comparable OTC benchmarks across cold-climate cities.

### Impact of sky view factor (SVF) on thermal comfort

The literature shows that SVF’s role is complex, with varying effects across climates. Simulations for a cold city (Montreal in Canada^57^, indicate that SVF is anticorrelated with OTC in summer and winter (i.e. increasing SVF improves OTC in summer but degrades OTC in winter). Another study in Beijing, China^58^ indicates that SVF < 0.3 tends to improve summer OTC but degrade winter OTC. By isolating SVF effects across seasons, we demonstrate that increased sky openness enhances comfort in winter but exacerbates heat stress in summer. Our study focused on SVF as a proxy for sky obstruction, but building height may also influence results. This inversion effect is critical for climate-sensitive design but has been underexamined in existing literature. Our model provides a quantifiable SVF–PET function, offering a dynamic tool for designers to modulate microclimates via canopy density, building height, and surface openness.

This study analyzed thermal comfort data for both winter and summer in Shenyang, confirming that the sky view factor (SVF) exerts a important and seasonally dependent influence on Physiological Equivalent Temperature (PET) and Universal Thermal Climate Index (UTCI). Specifically, lower winter SVF values (e.g., 0.500) were associated with reduced PET and UTCI values, since limited sky exposure decreases solar radiation, consequently diminishing thermal comfort. This trend is primarily observed for PET at SVF = 0.5. Conversely, lower summer SVF values (e.g., 0.073) corresponded to higher PET and UTCI values, as shading reduced direct radiation exposure, thereby improving comfort. This applies mainly to PET at SVF = 0.073. While our results suggest SVF effects, they are not robustly proven and should be compared with complex literature findings, such as those from^57^ Correlations do not imply causation; effects may be confounded by unmeasured variables.

This study contributes a novel insight into the ‘Seasonal Inversion’ of morphological impacts, which distinguishes the Severe Cold zone from other climates. Unlike studies in hot-humid regions (e.g., Singapore, Hong Kong) where shading (low SVF) is universally beneficial, or temperate regions where winter demand is negligible, our results reveal a critical design conflict in Shenyang. We found that the neutral PET range shifts by over 10 °C between seasons, and the impact of SVF reverses direction. This quantifies the necessity for ‘dynamic morphology’ (e.g., deciduous vegetation, adjustable shading) rather than static strategies, advancing the theoretical framework of climate-responsive design in high-latitude regions.

Future studies should investigate how humidity, wind speed, and vegetation types moderate the relationship between SVF and thermal comfort. Additionally, incorporating physiological indicators (e.g., skin temperature, heart rate) would elucidate underlying mechanisms and strengthen the scientific basis for climate-responsive urban design.

### Implications for urban design in severe cold zones

This study provides critical pathways for urban design to utilize the proposed adaptive comfort model, primarily through target-based space programming and SVF-responsive morphology. The determined neutral PET ranges (8.4–16.6 °C in winter and 19.1–26.0 °C in summer) serve as precise quantitative targets for outdoor space design, allowing designers to utilize microclimate simulation tools (e.g., ENVI-met) to verify if proposed plaza or park designs maintain thermal conditions within these locally calibrated benchmarks. Furthermore, addressing the seasonal conflict where high SVF is beneficial in winter for solar gain but detrimental in summer requires avoiding rigid designs like permanent solid roofs. Instead, the implementation of broad-leaved deciduous trees is recommended to naturally regulate SVF—providing shading in summer while maximizing solar access in winter—alongside adaptive infrastructure such as retractable awnings in high-density zones to manually adjust SVF according to the defined seasonal thresholds.

### Limitations

In summary, thermal comfort benchmarks (OTC) within Shenyang vary significantly due to variations in (1) data collection timing (seasonal vs. year-round), (2) measurement site types (squares, streets, parks, and pavilions), (3) statistical approaches (linear regression vs. probabilistic analysis), and (4) respondent demographics. This study provides seasonally differentiated, field-based OTC benchmarks for Dwa climate zones, establishing direct design thresholds for cold-climate urban planning regarding shading, wind protection, and vegetation strategies. We recommend SVF-based design thresholds, such as maintaining SVF below 0.7 in summer to reduce heat stress. Integrating acceptable thermal ranges into early planning stages enables architects to strategically incorporate water features, vegetation, and architectural forms that can extend comfortable outdoor activity durations to 6 h during summer daytime conditions. 6 h represents typical daytime outdoor activity duration in summer.

However, the study has several limitations: (1) The sample population was predominantly aged 18–32, lacking representation of older age groups who may exhibit different thermal adaptability; (2) The influence of activity type (such as walking, sitting, or exercising) on thermal perception was not distinguished; (3) Thermal comfort assessments were based solely on subjective questionnaires, without the inclusion of physiological indicators such as skin temperature or heart rate, and the potential role of multisensory interactions (e.g., thermal-olfactory effects) was not addressed. Future work should explore multisensory interactions, such as olfactory and sound effects on OTC. Future research should aim to expand the age range of participants, differentiate between specific activity scenarios, and integrate wearable physiological monitoring to uncover the coupled effects of multiple factors, thereby establishing a more inclusive and robust set of thermal comfort benchmarks for cold-climate urban environments.

While this study primarily focuses on thermal perception, it highlights the potential of integrating physiological data (e.g., skin temperature, heart rate) and multisensory interactions (e.g., light, sound, smell) in future research. These additions would further refine our understanding of human-environment interaction in harsh climates.

Finally, our seasonally stratified methodology addresses long-standing issues of bias in mixed-season datasets. By treating summer and winter as distinct behavioral and climatic regimes, this study presents a more accurate and transferable model for cold-region urban design. Future work should replicate this dual-season SVF framework across other Dwa and Dwb cities to test generalizability and expand its application scope.

## Conclusions

This study quantitatively investigated the seasonal dynamics of outdoor thermal comfort in the severe cold zone of Shenyang, specifically deciphering the conflicting roles of urban morphology. Based on 1,009 paired datasets, the following core conclusions are drawn:

(1) Distinct Seasonal Benchmarks: Residents exhibit strong physiological and psychological adaptation to the extreme climate. The Neutral PET threshold shifts significantly from 12.49 °C in winter to 22.54 °C in summer. These season-specific benchmarks provide more accurate compliance targets for local urban planning than annual aggregate models.

(2) The “SVF Inversion” Effect: Urban morphology exerts a seasonally opposing influence on thermal perception. High Sky View Factor (SVF) is identified as a critical heat source in winter (raising TSV) but a primary cause of heat stress in summer. This confirms that static morphological strategies valid in tropical regions (e.g., minimizing SVF) are inapplicable to Dwa climates.

(3) Implications for Adaptive Design: To reconcile this seasonal conflict, urban design in severe cold zones must prioritize “dynamic morphology.” We recommend the strategic use of deciduous vegetation and flexible shading infrastructure to maximize solar access in winter while providing necessary shading in summer.

In summary, this research clarifies the mechanism of seasonal adaptation in high-latitude cities and offers an empirically grounded framework for climate-responsive urban design in severe cold regions.

**Appendix 1**. Current research on outdoor thermal comfort in different climate regions of China.

**Table:** 

References	Location		Climatic zone	NPET (°C)	NPETR (°C)	NUTCI (°C)	NUTCIR (°C)	Season
This study	Shenyang, China		Dwa	22.5/12.5	19.1–26.0 /8.4–16.6	23.0/5.4	19.9–26.1 /1.6–9.2	Summer /Winter
Su et al., 2024^24^	Dalian, China		Dwa	20.8	16.9–24.6	24.5	20.6–25.8	Summer
Ma & Zhang, 2024^28^	Beijing, China		Dwa	21.9/14.3	15.2–28.6 /6.1–22.5			Summer /Winter
Chen et al., 2018^7^	Harbin, China		Dwa	20.0/18.0				Summer /Winter
Yin et al., 2021^23^	Harbin, China		Dwa	21.5/13.3(Summer /Winter)	13.0–21.0 /18.0–21.0 /11.0–24.0			All year /Summer /Winter
He et al., 2024^59^	Harbin, China		Dwa			13.3	1.4–25.2	Winter
Niu et al., 2020^60^	Xi’an, China		Dwa	26.1	20.0–32.1.0.1	27.6	24.0–31.3.0.3	Summer
Nikolopoulou & Lykoudis, 2006^46^	Tianjin, China		Dwa/BSk	15.6				All year
Lai et al., 2014^44^	Tianjin, China		Dwa/BSk		11.0–24.0	17.5	13.6–21.3	All year
Cheung & Jim, 2018^61^	Hong Kong, China		Cwa	21.3	18.9–31.6	22.7	19.9–33.1	Summer
Lam & Lau, 2018^22^	Hong Kong, China		Cwa			23.5	17.9–29.2	Summer
Lin, 2009^56^	Taiwan, China		Cwa	25.6/23.7				Summer /Winter
Lin & Matzarakis, 2008^62^	Taiwan, China		Cwa	27.2	26.0–30.0			All year
L. Chen et al., 2015^63^	Shanghai, China		Cfa		15.0–29.0			All year
Yang et al., 2013b^13^	Changsha, China		Cfa	28.1	24.0–31.0			Summer
Liu et al., 2016^64^	Changsha, China		Cfa	18.6	15.0–22.0			All year
J. Huang et al., 2016^2^	Wuhan, China		Cfa			19.2	11.1–27.4	All year
Fang et al., 2019^65^	Guangzho, China		Cfa			26.0		Summer
Zeng & Dong, 2015^66^	Chengdu, China		Cwa	24.4	26.1–30.0			Summer

**Appendix 2**. Current research on outdoor thermal comfort in different climate regions internationally.

**Table:** 

References	Location	Climatic zone	NPET (°C)	NPETR (°C)	NUTCI (°C)	NUTCIR (°C)	Season
Trindade Da Silva & Engel De Alvarez, 2015^67^	Vitoria, Brazil	Af		22.0–30.0 (All year)			Winter /spring /summer
Yang et al., 2013b^13^	Singapore	Af	28.1	24.0–30.0			Summer
Hirashima et al., 2016^14^	Bello Horizonte, Brazil	Aw	27.7/15.9	19.0–27.0 (All year)			Summer/Winter
Ndetto & Matzarakis, 2017^16^	Dar es Salaam, Tanzania	Aw	29 (All year)	23.0–31.5.0.5 (All year)			Cool/warm
Middel et al., 2016^18^	Tempe, Arizona	BWh	28.6	19.1–38.1			All year
Cohen et al., 2019^68^	Beer Sheva, Israel	BWh	22.8/20.6	17.0–26.0(All year)			Summer/Winter
Mahmoud, 2011^15^	Cairo, Egypt	BWh		22.0–30.1.0.1 /21.6–29.0			Summer/Winter
Hadianpour et al., 2018^17^	Tehran, Iran	BWk	21.5 /25.1 /21.9 /17.2	16.9–26 /22.1–28 /17.4–26.5 /13.9–20.5	20.8 /25.8 /21.5 /17.2	16.7–25.0 /23.5–28.1 /17.7–25.4 /14.2–20.1	Spring /Summer /Autumn /Winter
Yahia & Johansson, 2013^69^	Damascus, Syria	BSk		22.8–28.5 /15.8–21.0			Summer/Winter
Lucchese & Mikuri, 2016^70^	Campo Grande, Brazil	Cfa/Aw		21.0–27.0			Winter/Spring
Bröde et al., 2012^11^	Curitiba, Brazil	Cfa				18.0–23.0	All year
Sadeghi et al., 2018^71^	Sydney, Australia	Cfa	24.0				All year
Watanabe et al., 2014^72^	Nagoya, Japan	Cfa			34.0	32.2–35.9	Summer
Matzarakis et al., 1999^73^	Middle /western Europe	Cfb		18.0–23.0			
Kovács et al., 2016^74^	Szeged, Hungary	Cfb		13.1–20.3 /17.0–22.3.0.3 /14.7–22.6			Spring /summer /autumn
Lam & Lau, 2018^22^	Melbourne, Australia	Cfb			19.3		Summer
Shooshtarian & Ridley, 2016^75^	Melbourne, Australia	Cfb		14.0–28.0			Spring, summer, autumn
Kenawy & Elkadi, 2018^45^	Melbourne, Australia	Cfb	20.4 /21.4 /17.3	17.5–23.2 (All year)			All year /Summer /Winter
Krüger et al., 2013^21^	Glasgow, UK	Cfb		9.0–18.0			Summer
Katzschner, 2006^48^	Kassel, Germany	Cfb		18.0–21.0			All year
Pantavou et al., 2013^20^	Athens, Greece	Csa			20.3	16.3–24.2	All year
Salata et al., 2016^19^	Rome, Italy	Csa	26.9/24.9(Summer /Winter)	21.1–29.2 (All year)			All year /Summer /Winter
Cohen et al., 2013^55^	Tel Aviv, Israel	Csa	23.9/22.7(Summer/Winter)	20.0–25.0 (All year)			All year /Summer /Winter
Tsitoura et al., 2014^76^	Crete, Greece	Csa		20.0–25.0			Summer/Winter
Jeong et al., 2016^77^	Seoul, Korea	Dwa/Cwa		9.9–20.7			Summer/autumn
Lindner-Cendrowska & Błażejczyk, 2018^50^	Warsaw, Poland	Dfb		6.3–21.8			All year
B. Yang et al., 2017^78^	Umeå, Sweden	Dfc			14.4	11.5–17.2	Summer

**Appendix 3**. Neutral thresholds by residence duration and season.

**Table:** 

Season	Length of residence	Thermal index	Regression equation	Neutral temp. (℃)	Neutral range (℃)	*R*-squared	*P*-value
Summer	Overall	PET	MTSV = 0.1457 * PET − 3.2847	22.54	19.11–25.97	0.8771	8.2741e-14 *
		UTCI	MTSV = 0.1603 * UTCI − 3.6846	22.99	19.87–26.11	0.8892	5.4567e-12 *
	< 1 year	PET	MTSV = 0.1412 * PET − 2.9019	20.56	17.02–24.10	0.8842	5.1571e-05 *
		UTCI	MTSV = 0.2024 * UTCI − 5.0434	24.92	22.45–27.39	0.8345	5.7493e-04 *
	1–4 years	PET	MTSV = 0.1308 * PET − 2.8048	21.44	17.62–25.26	0.9393	1.2312e-13 *
		UTCI	MTSV = 0.1619 * UTCI − 3.7775	23.34	20.25–26.43	0.8389	5.7460e-09 *
	> 4 years	PET	MTSV = 0.1770 * PET − 4.3133	24.37	21.54–27.19	0.8425	4.6284e-09*
		UTCI	MTSV = 0.2149 * UTCI − 5.6119	26.11	23.79–28.44	0.8328	3.2791e-07 *
Winter	Overall	PET	MTSV = 0.1208 * PET − 1.5091	12.49	8.35–16.63	0.9149	3.6668e-12*
		UTCI	MTSV = 0.1313 * UTCI − 0.7050	5.37	1.56–9.17	0.8974	2.5267e-09 *
	< 1 year	PET	MTSV = 0.1474 * PET − 2.0381	13.83	10.44–17.22	0.8871	1.4810e-05 *
		UTCI	MTSV = 0.1204 * UTCI − 1.3821	11.48	7.32–15.63	0.7526	5.3979e-04 *
	1–4 years	PET	MTSV = 0.1045 * PET − 1.3353	12.78	8.00–17.57.00.57	0.7246	2.8907e-05 *
		UTCI	MTSV = 0.0927 * UTCI − 0.8198	8.85	3.45–14.24	0.5689	7.3637e-04*
	> 4 years	PET	MTSV = 0.1494 * PET − 1.4356	9.61	6.26–12.96	0.8201	5.0321e-05 *
		UTCI	MTSV = 0.1509 * UTCI − 0.5276	3.5	0.18–6.81	0.6105	4.5135e-03**​

**Appendix 4**. Seasonal SVF gradients versus PET: (a) summer scatter and (b) winter scatter.


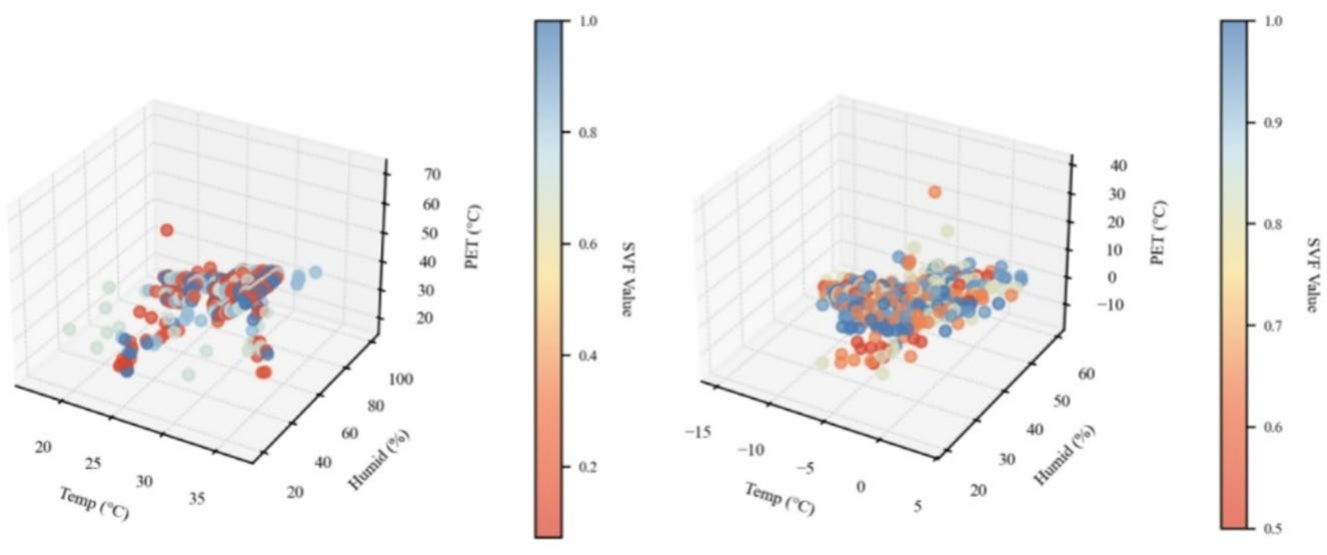


**Appendix 5**. Seasonal distribution of thermal comfort votes (TCV) across winter and summer samples.


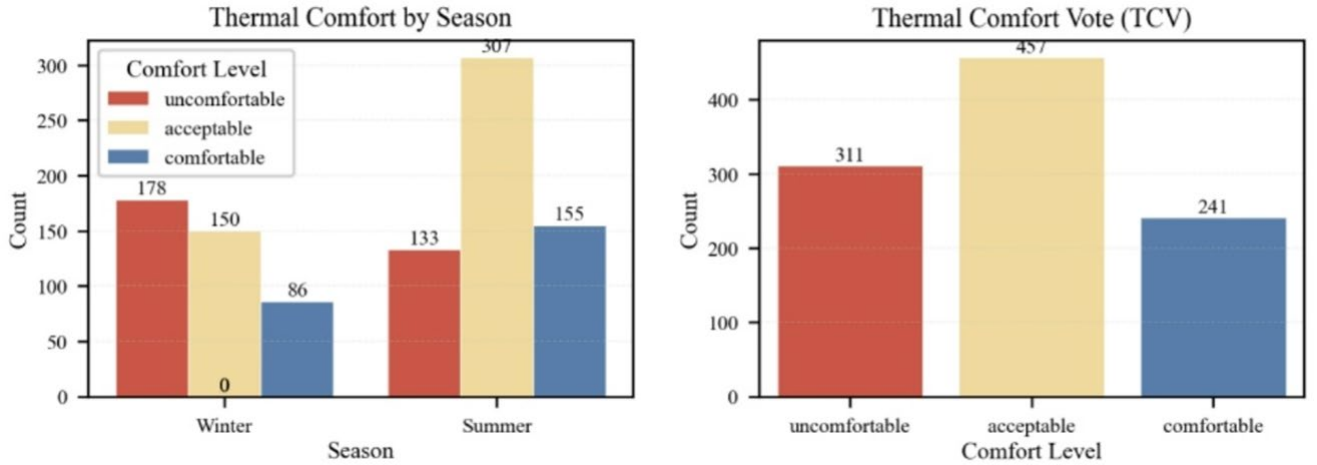


**Appendix 6**. Summer diurnal PET and UTCI evolution.


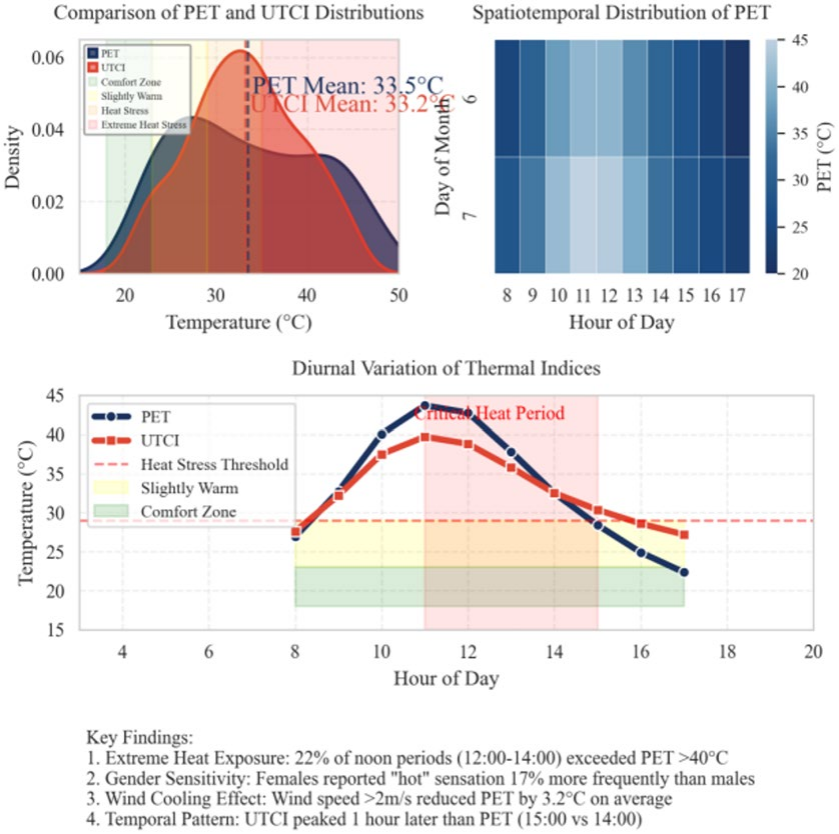


**Appendix 7**. Winter diurnal PET and UTCI evolution.


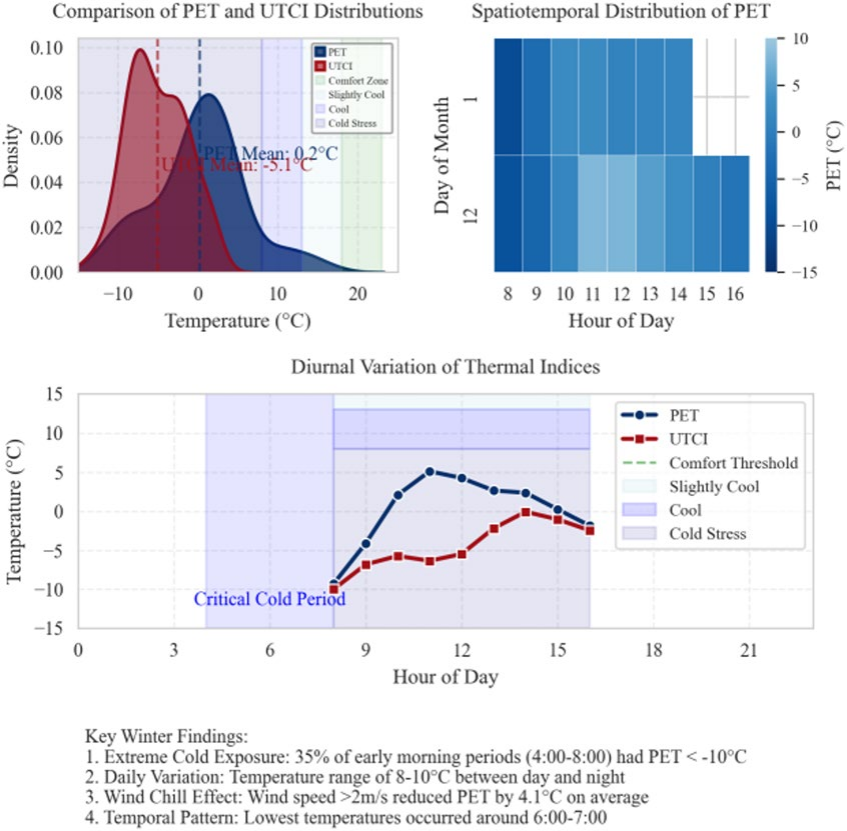


**Appendix 8**. Nomenclature and abbreviations.

**Table:** 

Abbreviation / Symbol	Full Term / Description
V/Va	Wind Speed (formerly denoted as Va​)
Ta​	Air Temperature
Tg​	Globe Temperature
Tmrt​	Mean Radiant Temperature
RH	Relative Humidity
SVF	Sky View Factor
LAI	Leaf Area Index
OTC	Outdoor Thermal Comfort
PET	Physiologically Equivalent Temperature
UTCI	Universal Thermal Climate Index
SET*	Standard Effective Temperature*
TSV	Thermal Sensation Vote
MTSV	Mean Thermal Sensation Vote
TCV	Thermal Comfort Vote
TPV	Thermal Preference Vote
NPET	Neutral PET (Neutral Temperature of PET)
NUTCI	Neutral UTCI (Neutral Temperature of UTCI)
NRPET	Neutral Temperature Range of PET
NRUTCI	Neutral Temperature Range of UTCI
Aw	Tropical, Savannah
BSh	Arid Steppe, Hot
BSk	Arid Steppe, Cold
BWk	Arid Desert, Cold
Cfa	Temperate without Dry Season, Hot Summer
Cfb	Temperate without Dry Season, Warm Summer
Csa	Temperate Dry Summer, Hot Summer
Cwa	Temperate Dry Winter, Hot Summer
Dfb	Cold without Dry Season, Warm Summer
Dwa	Cold, Dry Winter, Hot Summer

## Data Availability

Data is provided within the manuscript file.
